# Thermal Ablation as a Non-Surgical Alternative for Thyroid Nodules: A Review of Current Evidence

**DOI:** 10.3390/medicina61111910

**Published:** 2025-10-24

**Authors:** Andreas Antzoulas, Vasiliki Garantzioti, George S. Papadopoulos, Apostolos Panagopoulos, Vasileios Leivaditis, Dimitrios Litsas, Platon M. Dimopoulos, Levan Tchabashvili, Elias Liolis, Konstantinos Tasios, Panagiotis Leventis, Nikolaos Kornaros, Francesk Mulita

**Affiliations:** 1 Department of Surgery, General University Hospital of Patras, 265 04 Patras, Greece; a.antzoulas@hotmail.com (A.A.); vigarant@yahoo.com (V.G.); georgepapadop13@gmail.com (G.S.P.); tolioap12@gmail.com (A.P.); kostastasiosmd@gmail.com (K.T.); 2 Department of Cardiothoracic and Vascular Surgery, WestpfalzKlinikum, 67655 Kaiserslautern, Germany; vnleivaditis@gmail.com; 3 Department of Surgery, General Hospital of Lamia, 351 00 Lamia, Greece; dimlitsas@icloud.com; 4 Department of Radiology, General University Hospital of Patras, 265 04 Patras, Greece; dimopoylos.platonas@gmail.com; 5 Department of General Surgery, General Hospital of Eastern Achaia—Unit of Aigio, 251 00 Aigio, Greece; tchabashvili.alexander@gmail.com (L.T.); levedis6@gmail.com (P.L.); kornaros.nikolaos@hotmail.com (N.K.); 6 Department of Oncology, General University Hospital of Patras, 265 04 Patras, Greece; lioliselias@yahoo.gr

**Keywords:** thyroid nodule, image-guided ablation, radiofrequency ablation, microwave ablation, laser ablation

## Abstract

Thyroid nodules, prevalent in 2% to 65% of the general population depending on diagnostic methodology, represent a significant clinical concern despite a low malignancy rate, typically 1% to 5%. A substantial proportion of thyroid cancers are small, indolent lesions, allowing for conservative management with favorable prognoses. Nodule detection commonly occurs via palpation, clinical examination, or incidental radiological findings. Established risk factors include advanced age, female gender, obesity, metabolic syndrome, and estrogen dominance. Despite conservative management potential, a considerable number of thyroid nodules in Europe are unnecessarily referred for surgery, incurring unfavorable risk-to-benefit ratios and increased costs. Minimally invasive techniques (MITs), encompassing ethanol and thermal ablation modalities (e.g., laser, radiofrequency, microwave), offer outpatient, nonsurgical management for symptomatic or cosmetically concerning thyroid lesions. These procedures, performed under ultrasound guidance without general anesthesia, are associated with low complication rates. MITs effectively achieve substantial and sustained nodule volume reduction (57–77% at 5 years), correlating with improved local symptoms. Thermal ablation (TA) is particularly favored for solid thyroid lesions due to its precise and predictable tissue destruction. Optimal TA balances near-complete nodule eradication to prevent recurrence with careful preservation of adjacent anatomical structures to minimize complications. Radiofrequency ablation (RFA) is widely adopted, while microwave ablation (MWA) presents a promising alternative addressing RFA limitations. Percutaneous laser ablation (LA), an early image-guided thyroid ablation technique, remains a viable option for benign, hyperfunctioning, and malignant thyroid pathologies. This review comprehensively evaluates RFA, MWA, and LA for thyroid nodule treatment, assessing current evidence regarding their efficacy, safety, comparative outcomes, side effects, and outlining future research directions.

## 1. Introduction

Thyroid nodules are defined as discrete, radiologically distinct lesions within the thyroid parenchyma, exhibiting variable cytological and sonographic characteristics, biological behavior, and clinical relevance; while the majority are benign and indolent, a subset may carry malignant potential and warrant further diagnostic evaluation or therapeutic intervention based on individualized risk assessment [1]. The prevalence of thyroid nodules within the general population ranges from 2% to 65%, with variations attributed to the specific diagnostic methods employed [2]. Despite the concern regarding malignancy, the actual prevalence of thyroid cancer within unselected nodule populations generally ranges from 1 to 5%; this rate is subject to variation influenced by selection criteria and the specific demographic characteristics of the evaluated population, such as the inclusion or exclusion of papillary microcarcinomas [3]. When diagnosed with thyroid cancer, a significant proportion of cases involve small, intra-thyroidal neoplasms with an indolent growth pattern. As demonstrated by a recent large-scale study of an unselected population, up to 53.6% of thyroid cancers fall into this category [4]. Consequently, many cases can be managed conservatively with a favorable prognosis [5]. Thyroid nodules are typically detected through various methods, including self-palpation by patients, clinical examination during routine health checkups, or incidental findings on radiological imaging procedures such as ultrasonography, computed tomography, magnetic resonance imaging, or fluorodeoxyglucose positron emission tomography performed for other diagnostic purposes [6]. Several risk factors have been identified for the development of thyroid nodules. Advanced age, female gender, and obesity have been consistently associated with an increased susceptibility to these lesions [7]. Furthermore, research suggests a correlation between the metabolic syndrome and its components and the prevalence of thyroid nodules [8]. Additionally, estrogen dominance has been proposed as a potential contributing factor or causative agent in the development of thyroid nodules [9]. Within European countries, a portion of thyroid nodules are unnecessarily referred for surgical intervention, resulting in an unfavorable risk-to-benefit ratio and increased costs [10]. A more conservative management approach can effectively moderate the risk of complications and the associated financial burden. Radioiodine (^131^I) therapy represents a well-established nonsurgical option for autonomously functioning (hot) thyroid nodules, achieving normalization of thyroid function in most patients and significant but gradual nodule shrinkage, although with a long-term risk of hypothyroidism [11]. The implementation of minimally invasive techniques (MITs) for the management of thyroid nodules has been limited and remains insufficiently evaluated in clinical practice [12]. These ultrasound-guided, outpatient procedures offer a nonsurgical alternative for treating thyroid lesions that produce compressive symptoms or aesthetic concerns [13]. MITs comprise ethanol ablation (EA) and thermal ablation (TA) modalities, which employ various energy sources, including laser, radiofrequency, microwave, and high-intensity focused ultrasound. These interventions are typically performed without the need for general anesthesia, and the incidence of complications is low [14]. Clinical studies have demonstrated a substantial and durable reduction in nodule volume, with reported decreases ranging from 57% to 77% at five years post-procedure [15]. This reduction is often accompanied by an improvement in local symptoms [16]. Thermal ablation (TA) procedures, owing to their precise and predictable destruction of tissue volumes, are considered the primary treatment option for solid thyroid lesions [14]. Thermal ablation (TA) treatments should aim to achieve an optimal balance between sufficient nodule volume reduction to prevent regrowth and the preservation of adjacent critical anatomical structures, thereby minimizing the risk of complications [17]. Radiofrequency ablation (RFA) is the most widely employed technique, although microwave ablation (MWA) represents a promising alternative with the potential to address certain limitations of RFA [18,19]. Percutaneous laser ablation (LA) was among the earliest techniques employed for image-guided thyroid ablation (IGTA) and remains a viable option in various clinical settings [20]. Its minimally invasive nature and precise application offer potential advantages over other ablative modalities [21]. LA has also been historically utilized for thyroid thermal ablation, with reported applications in treating benign, hyperfunctioning, and malignant thyroid pathologies [22,23]. This review aims to provide a comprehensive evaluation of thermal ablation techniques, including radiofrequency ablation (RFA), microwave ablation (MWA), and laser ablation, for the treatment of thyroid nodules. The review will assess the current evidence supporting the efficacy and safety of these techniques, compare their outcomes and side effects, and identify areas for future research.

## 2. Methods

**Figure 1:**
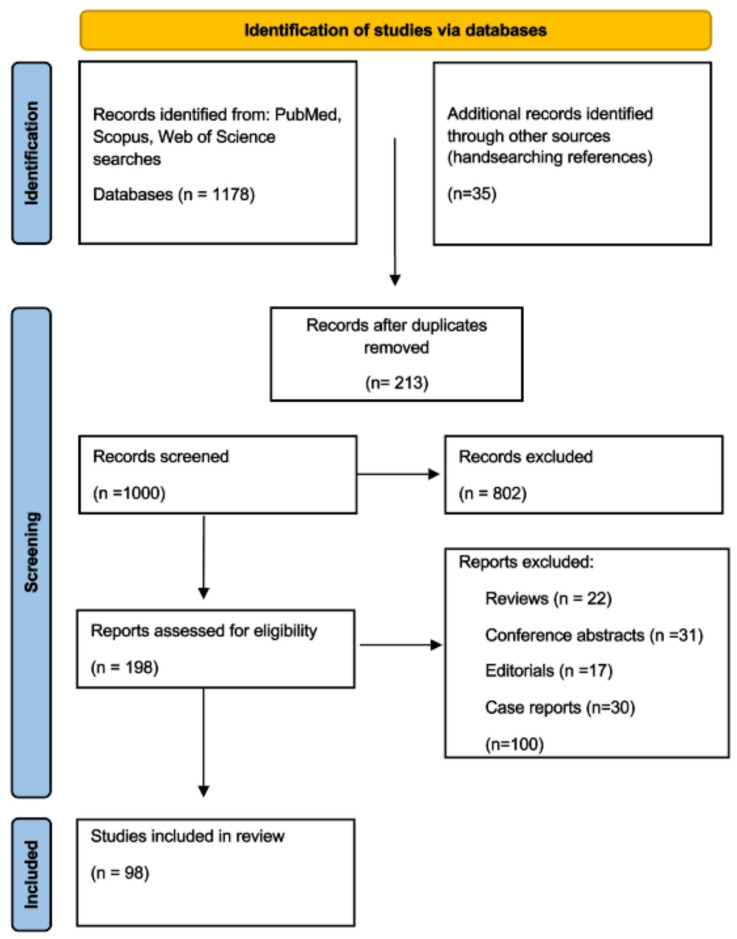
Flowchart of the study selection process.

This narrative review was conducted to summarize and critically appraise the current evidence on thermal ablation as a non-surgical alternative for thyroid nodules. A comprehensive literature search was performed in PubMed, Scopus, and Web of Science for studies published between January 2000 and September 2025. The following search terms were used: “thermal ablation” OR “radiofrequency ablation” OR “microwave ablation” OR “laser ablation” combined with “thyroid nodule” OR “thyroid tumor” OR “thyroid neoplasm”. All peer-reviewed original articles, clinical trials, cohort studies, and large case series reporting clinical outcomes of thermal ablation for thyroid nodules were considered eligible. Only studies published in English were included. Reviews, conference abstracts, editorials, and single case reports with fewer than 10 patients were excluded. The search and selection process identified 98 eligible studies, which were included in the final narrative synthesis. Due to heterogeneity in study design, patient populations, and outcome measures, no quantitative meta-analysis was performed. Instead, findings were synthesized narratively, with emphasis on treatment efficacy, safety, long-term outcomes, and comparative advantages of different thermal ablation modalities. The Flowchart illustrates the study selection process (Figure 1).

## 3. Radiofrequency Ablation (RFA)

### 3.1. Basic Principles of RF Ablation

Globally, radiofrequency ablation (RFA) has been utilized as a clinical modality for over two decades. The initial human experience with RFA for the management of benign thyroid nodules was described in the literature by Kim et al. [24]. Following its initial application, the technology experienced broad dissemination throughout Asia and Europe. The subsequent development of clinical guidelines and consensus statements in these regions further established RFA as a valuable element within individualized treatment strategies and broadened its clinical indications beyond benign pathologies [14,25]. The minimally invasive nature of radiofrequency ablation (RFA) obviates the necessity for general anesthesia, avoids the sequelae of a cervical scar, and reduces the duration of the recovery period. However, its principal advantage over surgical intervention lies in the preservation of euthyroidism [26]. While transient thyroid dysfunction may be observed post-RFA, the long-term maintenance of normal thyroid function represents a key differentiating factor [27]. In essence, radiofrequency ablation (RFA) employs thermal energy generated by high-frequency alternating electric current oscillating within a range of 200 to 1200 kHz [28]. The passage of radiofrequency (RF) waves through the electrode induces agitation of tissue ions in the immediate vicinity, leading to a localized increase in temperature within the tumor tissue Via frictional heating. This thermal energy results in the destruction of neoplastic cells situated within a few millimeters of the active electrode. Furthermore, thermal conduction from the primary ablation zone can cause a comparatively slower rate of cellular damage in more distal tumor regions or adjacent tissues. This dual process of thermal injury, mediated by both frictional and conductive heat transfer, constitutes the fundamental mechanism of radiofrequency ablation [29]. Within the temperature range of 60 to 100 °C, rapid tissue coagulation occurs, leading to irreversible cellular damage in the tumor. Conversely, temperatures exceeding 100–110 °C induce tissue vaporization and carbonization, which can act as a thermal insulator, thereby impeding further heat propagation and consequently diminishing the efficacy of radiofrequency ablation [30]. Maintaining intratumoral temperatures between 50 °C and 100 °C is critical for effective treatment [31]. Fibrotic or calcified tissues, characterized by low ionic content, require higher energy delivery for adequate heat generation. Tissue perfusion contributes to heat dissipation via a heat-sink effect from intratumoral or perinodular vasculature [32], reducing therapeutic efficacy in hypervascular nodules, which necessitates the application of increased RF energy [31].

### 3.2. Patient Selection, Pre-Procedural Evaluation, and Treatment Planning

Judicious patient selection for thyroid RFA necessitates high confidence in nodule benignity, typically evidenced by two benign fine-needle aspiration biopsies or one benign biopsy with a classically benign ultrasound appearance (spongiform or predominantly cystic) [25,33]. Symptomatic nodules, particularly larger ones contributing to compressive or aesthetic concerns, are primary candidates, although no strict size minimum exists [14]. While size criteria vary across guidelines (e.g., >2 cm with growth, >20 mL), the unifying principle is symptom relevance relative to nodule location and patient habitus. A collaborative decision-making process between patient and physician is crucial, potentially involving symptom and cosmetic disturbance quantification [34]. Ideal candidates present with a well-defined, dominant cervical nodule directly correlating with reported symptoms [35]. Multinodular goiter and substernal nodules warrant cautious consideration, as RFA is not currently first-line for these conditions, although emerging evidence suggests potential utility [36]. Ablation of consistently growing smaller nodules may also be considered for preemptive treatment [37]. Ultimately, patient motivation and realistic expectations are paramount for successful outcomes. For autonomously functioning thyroid nodules (AFTNs), RFA aims to restore euthyroidism and discontinue medication, with higher success rates in smaller nodules (<10 mL) [38]. Both radiofrequency ablation (RFA) and radioactive iodine (RAI) are effective options for autonomously functioning thyroid nodules (AFTNs). While RAI remains the standard non-surgical treatment with reliable hormonal control, it may cause hypothyroidism or be unsuitable for some patients. RFA offers a radiation-free alternative that preserves thyroid tissue but is generally less effective in restoring euthyroidism. Therefore, RFA is best reserved for patients who decline or have contraindications to surgery or RAI [39]. Pre-procedural evaluation for thyroid RFA necessitates a comprehensive ultrasound (US) assessment to characterize nodule features (size, shape, margin, composition, echogenicity, calcification, vascularity, extracapsular invasion) and surrounding anatomy [40]. Nodule volume should be calculated using three orthogonal US measurements via the formula: V = πabc/6. Standard laboratory evaluation includes complete blood count, coagulation profile, thyrotropin, thyroid hormones, thyroid autoantibodies, and calcitonin levels. Any detected abnormalities, such as coagulation disorders, uncontrolled thyroid dysfunction, or other metabolic imbalances, should be appropriately managed and stabilized before proceeding with ablation to minimize procedural risks and ensure patient safety. Pre-ablation US is critical for evaluating recurrent tumor size, characteristics, and adjacent vital structures. Neck computed tomography (CT) may be indicated for further assessment of recurrent tumors, particularly when ultrasound findings are inconclusive or when there is suspicion of extrathyroidal extension, involvement of adjacent structures, or metastatic lymph nodes [41].

### 3.3. RFA Technique

Following adequate local anesthesia and hydrodissection to protect critical adjacent structures, the radiofrequency ablation (RFA) electrode is introduced through a midline cervical skin puncture. A “transisthmic approach”, involving passage through the thyroid isthmus, is typically employed to access the thyroid nodule. This approach provides a buffer of normal thyroid tissue, mitigating the risk of thermal injury to superficial tissues during ablation. Ablation commences at the caudal (inferior) aspect of the target nodule. Utilizing the “moving-shot technique”, the electrode is sequentially inserted into the posteromedial aspect of each axial plane of the nodule and then retracted in a posterolateral to anteromedial trajectory. The initiation of ablation is characterized by the appearance of hyperechoic microbubbles emanating from the active electrode tip under ultrasonographic guidance. The proceduralist employs real-time ultrasound visualization of the evolving ablated tissue and monitors tissue impedance displayed on the RFA generator console to determine the adequacy of energy delivery to each specific tissue volume. Once a complete axial plane has been ablated, the ultrasound transducer is repositioned to visualize a more cranial (superior) axial plane, and the ablation process is repeated in a caudal-to-cranial direction until the entire nodule has undergone treatment. Larger nodules may necessitate multiple skin punctures to ensure comprehensive access to superior aspects. Given that patients remain conscious during the procedure, their direct feedback regarding any discomfort or unusual sensations is crucial. The proceduralist must heed patient reports, as increasing discomfort may indicate excessive heat transfer to adjacent critical structures, such as the tracheal cartilages. When ablating in proximity to the trachea, the use of low power settings and/or smaller active tip electrodes is recommended to minimize the risk of inadvertent thermal injury. Upon completion of the ablation, the electrode is carefully withdrawn, and manual pressure, with or without the application of ice, is applied to the neck for a duration of 30 to 60 min to minimize post-procedural bruising and hematoma formation.

### 3.4. Outcomes

The evaluation of radiofrequency ablation efficacy is predicated on the measurement of nodule volume change and the documented improvement in patient-reported or clinically observed issues, notably pressure-related symptoms and cosmetic appearance. Following radiofrequency ablation, nodule volume reduction is a time-dependent process. An initial inflammatory response in the first week post-procedure may result in a transient increase in nodule volume, ranging from 0% to 20% of the baseline measurement, and the most significant volumetric decrease is typically observed within the initial six months [26]. Subsequent, albeit less pronounced, volume reductions may continue for a period of up to two to three years following the intervention. A meta-analysis by Cesareo et al. [34] quantified the aggregate effect of radiofrequency ablation on nodule volume, reporting mean reductions of 64.5% at 6 months, 76.9% at 1 year, and 92.2% at 3 years post-treatment. Furthermore, and perhaps of paramount importance, radiofrequency ablation demonstrates high efficacy in the management of symptomatic and cosmetic sequelae associated with thyroid nodular disease, leading to improvements in patient-reported quality of life [42]. The baseline volume of a thyroid nodule influences the final residual volume achieved after maximal shrinkage. Notably, nodules with an initial volume greater than 20 mL are associated with an increased likelihood of requiring multiple ablation sessions to attain satisfactory volume reduction [43]. Conversely, thyroid nodules with an initial volume below 10 mL are more likely to achieve a volume reduction ratio (VRR) of ≥80%, a threshold associated with thyrotropin normalization at 12 months post-procedure [3]. Similarly, the intrinsic composition of a nodule influences treatment response; nodules with cystic or predominantly cystic features generally experience faster and greater volume reduction compared to solid nodules [44]. Notably, a prospective investigation conducted in Italy reported the highest mean VRR (76%) in nodules characterized by a microcystic spongiform pattern on ultrasonography, whereas solid nodules exhibited an average volume reduction of 67%, these findings underscore the role of nodule morphology in predicting the therapeutic response to radiofrequency ablation [45]. Subsequent therapeutic intervention is frequently indicated in cases of nodule recurrence following initial radiofrequency ablation. Recurrence is commonly defined as a volumetric increase of 50% or greater relative to the nadir nodule volume previously documented [46]. The etiological factors contributing to regrowth are multifaceted and intrinsically linked to the technical efficacy of the initial ablation procedure. The predominant mechanism of regrowth typically involves the proliferation of residual, untreated peripheral nodular tissue that maintains its vascular perfusion [47]. Monpeyssen et al.’s [48] recent systematic review of 933 nodules reported a variable rate of nodule regrowth, ranging from 0% to 34% at the 12-month follow-up. Sim et al. [47] conducted a study involving 54 patients with thyroid nodules, followed for a mean duration of 39.4 months. The results of this investigation indicated that 24.1% of the treated nodules exhibited regrowth, while a more substantial proportion, 57.4%, demonstrated a clinically significant increase in volume.

### 3.5. RFA Complications

Several complications associated with thyroid radiofrequency ablation have been documented, including pain, voice alteration, hematoma formation, skin burn at the puncture site, thyrotoxicosis, hypothyroidism, edema, and fever. However, the majority of patients experienced resolution of these complications without long-term sequelae [49]. Pain during or after the procedure is a complication experienced by a significant minority of patients, with studies reporting rates as high as 21% [50]. During ablation, damage to the recurrent laryngeal or vagus nerves can result from two primary mechanisms: direct thermal injury due to unintended heat conduction from the electrode, or mechanical compression caused by intranodular hemorrhage. The majority of thermal neural injuries are reported to be transient, with voice alterations persisting for a period ranging from several weeks to months [51]. Hematoma formation, which is generally self-limiting, constitutes a common risk in ablation procedures. The etiology of these hematomas is frequently attributed to the disruption of pericapsular thyroid veins, which can occur during the introduction and maneuvering of the electrode [52]. Nodule rupture, characterized by the acute onset of neck bulging and associated pain during the post-ablation follow-up period, is a consequence of rapid nodule volume expansion resulting from hemorrhage [51]. The possibility of nodule regrowth, potentially requiring a subsequent ablative intervention, represents another outcome that should be communicated to patients. A key factor influencing the occurrence of regrowth is the patient’s baseline nodule volume, with larger initial volumes conferring a higher risk [53].

## 4. Microwave Ablation (MWA)

### 4.1. Principles of Microwave Ablation and Inclusion Criteria

Percutaneous microwave ablation (PMWA) induces molecular rotation using high-frequency microwave energy (2450 MHz), which increases local kinetic energy. This leads to a rise in tissue temperature, ultimately resulting in targeted tissue ablation [54,55]. The extent of thermal damage from microwave ablation depends on both the duration of exposure and the temperature reached within the tissue [56]. According to the 2020 European Thyroid Association Clinical Practice Guidelines—based on low-quality evidence—MWA is considered a second-line thermal ablation technique. It is recommended for patients who are unsuitable for, decline, or are participating in clinical studies involving other thermal ablation modalities [14]. MWA is indicated for benign thyroid nodules larger than 4 cm, those causing compressive symptoms (such as discomfort, pain, or a foreign body sensation in the neck), cosmetic concerns, and in malignancies where surgery is contraindicated or refused [54,55]. To confirm a benign diagnosis, it is recommended to perform at least two ultrasound-guided fine-needle aspirations or core biopsies before initiating therapy. Ultrasound is also essential for characterizing nodules and evaluating adjacent anatomical structures. Routine pre-procedural laboratory tests should include: complete blood count, electrolytes, coagulation panel, calcitonin, TSH, free T4 (fT4), and triiodothyronine (T3). Fiberoptic laryngoscopy is advised for all patients before the procedure to assess baseline vocal cord function and identify any pre-existing laryngeal abnormalities, and it should be repeated afterward if hoarseness develops, as this allows prompt detection and management of potential recurrent laryngeal nerve injury [56,57].

### 4.2. MWA Devices and Procedures

Required materials include a cooled-shaft antenna, a microwave generator, and a flexible low-loss coaxial cable. The antenna, made of polytetrafluoroethylene (to prevent tissue adhesion), is a 16G needle with a 10 cm shaft and a 3–5 mm active tip. It is internally cooled with circulating distilled water. The generator delivers energy either in pulses or continuously, generating 1–100 W at 2450 MHz [54,58]. The patient is placed in a supine position with neck extension. A venous catheter is inserted, local antisepsis is performed, and 2% lidocaine is administered subcutaneously for local anesthesia. The “liquid-isolating region” technique may be used, wherein physiological saline mixed with 0.9% lidocaine is infused into the thyroid capsule to protect surrounding vital structures from thermal injury. The antenna is inserted along the long axis of the nodule under ultrasound (US) guidance. Typically, 30–50 W of energy is delivered over 5–15 min. The ablation zone appears hyperechoic on US. If this does not occur within 5–10 s, the power is increased by 5 W. For mixed or predominantly cystic nodules, internal fluid should be aspirated before ablation. The procedure is terminated once the entire nodule is covered by a hyperechoic zone. To ensure complete ablation, repositioning of the antenna tip (the “moving shot” technique) is often necessary. Hydrodissection may be used to increase the distance between the lesion and adjacent critical structures [54,56,58]. The average price for the device is 20,000–25,000 EUR, for the antenna 1000–1250 EUR, and for the MWA applicators about 6000 EUR [14].

### 4.3. MWA Clinical Results and Follow-Up Evaluation

Treatment efficacy is primarily measured by the reduction in nodule volume and improvement of related symptoms, including cosmetic concerns and compressive effects [56]. While high-quality evidence is limited, existing data suggest that both RFA and MWA significantly reduce thyroid nodule volume, with no statistically significant difference between the two [59,60]. Similarly, no clinically meaningful differences have been noted between MWA and surgery, although the evidence remains of low quality [61,62]. Across the literature, reported volume reduction for benign solid thyroid nodules after MWA ranges from 45% to 65% at up to 12 months of follow-up [63,64]. Mixed or predominantly cystic nodules generally show greater volume reduction than solid ones, likely due to the aspiration of cystic contents before ablation. Thyroid function may be slightly affected: a mild decrease in TSH, an increase in fT4, and no significant change in triiodothyronine levels have been reported one day after MWA [56,65,66]. In one study, Liu et al. reported a mean volume reduction of 90%, with some nodules achieving up to 94% reduction [67]. Regrowth of nodules can occur over time, and repeat ablation or surgery may be necessary in such cases [14]. Patients should undergo ultrasound examinations with Doppler at 1, 3, 6, and 12 months post-ablation to assess changes in nodule size, echogenicity, and vascularity, as altered blood flow may indicate residual or recurrent tissue. In addition, if symptoms of thyroid dysfunction arise, appropriate laboratory tests—including thyroglobulin (Tg) and thyroid antibodies (anti-TgAb, anti-TPOAb, and TRAb)—should be performed to monitor thyroid function, autoimmune activity, and treatment response. Clinical signs and symptoms should also be evaluated at the end of the procedure and again at one month to assess for complications or side effects [56].

### 4.4. MWA Complications

MWA carries a higher complication rate compared to LTA and RFA, likely due to the larger bore applicator used [14]. However, MWA has shown a clinically significant benefit over surgery regarding postoperative pain, although the supporting evidence is of low quality [61,62]. Reported complications include: hematomas, thyroid dysfunction, transient voice changes, recurrent laryngeal nerve injury, local pain, and skin burns. Post-procedure neck compression is sometimes recommended to prevent hematoma formation [54,63]. Voice changes are usually temporary and are attributed to thermal injury to the recurrent laryngeal nerve or nerve compression from perinodular edema. Identified risk factors for nerve injury include short distances between the nodule and the posterior capsule, as well as proximity to the tracheoesophageal groove [68]. To minimize risk, several techniques have been proposed, including the moving-shot technique, avoiding complete ablation near nerve zones, staged ablations for bilateral nodules, trans-isthmic approach, thermocouple needle monitoring, and the Liquid-isolation technique. Although no fatal complications have been reported, serious adverse events—such as esophageal perforation, tracheal injury, infection, and abscess formation—are theoretically possible due to the complex anatomy of the area [56].

## 5. Laser Ablation (LA)

### 5.1. Principles and Inclusion Criteria

Many are familiar with the term laser as a way to treat skin and eye disorders. However, under similar functionality, it can be beneficial for nodule removal. Laser ablation for thyroid nodules was first introduced in 2000, when Pacella et al. evaluated the use of an alternative treatment for recurrent thyroid carcinoma after surgery or radioiodine administration. They suggested that laser ablation could be a treatment plan for benign thyroid nodules [69]. Later on, in 2010, the American Association of Clinical Endocrinologists, the Italian Association of Clinical Endocrinologists, and the European Thyroid Association recommended that laser ablation is a possible and effective choice for benign nodules [70]. Laser, an acronym for Light Amplified Stimulated Emission of Radiation, is a coherent, monochromatic, collimated light energy that can target precisely the defined area through an optical fiber. Most commonly, diode or neodymium–yttrium aluminum garnet (Nd:YAG) are used to generate the light energy, at 820 nm or 1.064 nm wavelengths, respectively. The optical fiber can be cooled throughout its course to prevent any heating damage proximal to the targeted area [14,32,71,72]. When the hit occurs, a rapid thermal energy transfer causes permanent damage. The tissue is destroyed by energy absorption and can continue for up to 72 h more, due to ischemia and coagulation of the microvessels [32]. Depending on the temperature, the injury can vary from coagulative necrosis (46–100 °C) to tissue carbonization and vaporization (100–110 °C) [5]. However, temperatures over 100 °C may hamper the complete ablation of the targeted tissue [71]. Laser ablation is considered a viable option for benign thyroid nodules, such as cold nodules, local pressure symptoms, pain, neck discomfort, cosmetic concerns, normal TSH and FT4 values, and for those who are not eligible or refuse surgery or RAI therapy. It is also performed to de-bulk unrespectable masses and nodules close to critical structures. Small and hyperfunctioning thyroid nodules could also benefit from laser ablation [14,32,69,71,73,74].

### 5.2. LA Devices and Procedures

Laser ablation is performed as an outpatient procedure on fasting patients. As mentioned, most of the equipment in use consists of diode lasers operating from 800 to 980 nm, and lasers with Nd:YAG crystals at 1064 nm. The operation power has been described to range from 2 to 7 W. Usually, a fixed-power protocol of 3 W is used, with an illumination time from 400 to 600 s, in which each optical fiber produces 1200–1800 Joules of energy [70]. Various numbers of quartz optical fibers are inserted 1–4, depending on the nodule’s volume, with a 10 mm distance from one another, into the targeted nodule through thin and flexible 21-gauge needles under continuous ultrasonic guidance [71]. The preferred entry point is the isthmus, along its longer axis, but this can vary according to the exact location of the nodule. The tip of the needle is guided into the deepest part of the nodule, with a distance of 15 mm from the inferior margin and the surrounding structures [72]. Then, the optical fibers are forwarded to the nodule and are withdrawn 5–10 mm so only the tip is in direct contact with the nodule. Their diameter usually is 300 μm but can range up to 600 μm. The fibers are placed in a way to form an ellipsoid shape, matching most of the thyroid nodules [73]. Small nodules, less than 5 mm may need only one fiber for a short duration of the session, while larger ones with a mean width, thickness, and length at 40–50 mm, 30–35 mm, and 50–70 mm, respectively, may require a higher dose of energy, multiple fibers, and the usage of pullback technique [74,75]. This technique allows for to withdrawal of the fibers by 1–1.5 cm and repositioning them. Then, additional energy was given, which can lead to further destruction of large nodules. The duration of the illumination depends on the nodule’s size and can be in the range of 6 to 30 min. The patient is positioned supine with neck hyperextension. Local anesthesia is administered at the entry site [76]. It consists of 2% Lidocaine given subcutaneously, followed by subcapsular infiltration of Lidocaine (2–5 mL) under ultrasound guidance. It is preferred that the patient remain awake, under light sedation, in order to complain about any pain that occurs during the ablation. This helps prevent unnecessary trauma and complications to the surrounding structures [77].

### 5.3. Clinical Results and Follow-Up Evaluation

Rahal et al. [76], in a one-year follow-up of 30 patients, estimated a volume reduction of 60%. Only 10% complained about minor side effects. 97% responded well to the ablation, while one patient (3%) reported only a slight improvement. Pacella et al. [78], in a multicenter study of 1531 patients from 8 different thyroid centers, reported a mean reduction of 72% with an 11% deviation in nodule volume. There was also improvement in local symptoms and cosmetic signs. Minor and major complications occurred in only 0.9% combined. Dossing et al. [79] in a 10-year follow-up evaluation, reported a median reduction of 51% in solitary benign thyroid nodules of 78 patients. 84% and 72% of them claimed an improvement in symptoms and cosmetic complaints, respectively. Only 33% had minor complications, while none presented with major side effects. Gambelunghe et al. [80] also published results of a 10-year follow-up, which indicated a volume reduction ratio of 68% and 59% at 1 year and 10 years, respectively, after percutaneous ultrasound-guided laser ablation. 89% reported symptom improvement, while 81% showed cosmetic improvement. 98% described the procedure as well-tolerated. They also found an association between the amount of energy used and the development of minor complications. No major compilation was reported. 1.7% presented a nodule regrowth after 4 years and 4.7% at 7 years. Valcavi et al. [81] in a three-year study of 122 patients with cold nodules achieved a volume reduction of 47.8% with a deviation of 33.1%, symptoms improvement of 73%, and cosmetic improvement of 71.3%. The patients are observed for 2 h in a recovery room. Afterward, an ultrasonic examination is performed, and they are ready to leave [32,73]. Follow-up examinations are performed after 3, 6, and 12 months. They consist of a physical examination, ultrasound with Doppler, TSH and FT4 count, and antibodies count (Tg, antiTgAb, anti-TPOAb, and TRAb). Calcitonin should also be reviewed prior to the LA, and 1 year after [17,75,76]. However, if any complication occurs, an imaging examination should be performed immediately [78].

## 6. Thermal Ablation–Associated Complications

Thermal ablation procedures for thyroid nodules are generally safe, but several complications have been reported across radiofrequency ablation (RFA), microwave ablation (MWA), and laser ablation (LTA) techniques. Common adverse events include pain, voice alterations, hematoma formation, skin burns at the puncture site, edema, fever, and thyroid dysfunction, which may manifest as either hyper- or hypothyroidism [14,32,49,50,72,73,78,80,81]. Pain, the most frequently reported minor side effect, may radiate to the jaw, chest, shoulders, back, or ears and occurs in up to 21% of cases [82,83,84,85]. Voice changes, often resulting from thermal injury or nerve compression, are typically transient and resolve within weeks to months [51,70,73,86,87,88]. Hematomas, largely self-limiting, may result from pericapsular vessel injury during needle placement, particularly with larger gauge needles such as G22–27 [32,52,73,78,84,85,89,90,91,92]. Intranodular bleeding, pericapsular bleeding, and pseudocyst formation have also been observed [14,32,73,78,81,84,88,91]. Nodule rupture, presenting with acute neck swelling and pain, is another potential post-ablation event [51]. Though rare, stridor and cough have been described as minor complications [32,73,81]. MWA, due to its larger bore applicator, may be associated with a slightly higher complication rate compared to RFA and LTA [14]. Major complications such as vocal cord palsy, attributed to nerve injury or perineural inflammation, have been documented [70,72,73,76,77,78]. Risk factors for neural injury include close proximity of the nodule to the posterior capsule or tracheoesophageal groove [68]. Techniques such as the moving-shot technique, liquid-isolation, trans-isthmic approach, staged ablations, and thermocouple monitoring have been proposed to minimize these risks [56,68]. While serious complications like esophageal perforation, tracheal injury, infection, and abscess formation are theoretically possible due to the intricate cervical anatomy, no fatal complications have been reported to date [56]. The overall complication rate remains low, with large multicenter studies reporting combined rates of approximately 0.9% [78]. Additionally, nodule regrowth—often linked to larger baseline volumes—may necessitate subsequent treatment [53].

## 7. Qualification for Thermal Ablation

Recent advances in thermal ablation (TA) for thyroid nodules emphasize the importance of refined patient selection strategies based on ultrasound (US) parameters and the integration of artificial intelligence (AI) to improve treatment outcomes. High-resolution ultrasound remains the primary modality for evaluating eligibility, with key selection criteria including nodule size (<3–4 cm for solid nodules), composition (solid, mixed, or cystic), echogenicity, vascularity, well-defined margins, and the absence of proximity to critical structures such as the trachea or recurrent laryngeal nerve. Cytological confirmation by fine needle aspiration cytology (FNAC) is also essential, with most guidelines recommending at least a benign result (Bethesda category II) before removal [93]. In selected cases with indeterminate cytology (Bethesda III–IV), removal may be considered when surgery is contraindicated or refused, although this remains controversial and usually requires additional risk stratification. Several studies have demonstrated that specific ultrasound characteristics are predictive of favorable post-ablation response; for example, isoechoic, mixed solid-cystic, and hypervascular nodules are associated with significantly higher volume reduction rates (VRR) compared to purely solid or hypoechoic nodules [94]. In contrast, nodules with dorsal positioning, high vascularity, or large volume are more likely to be associated with incomplete ablation and complications [95]. Beyond traditional imaging evaluation, recent machine learning models have been developed to support clinical decision-making. Agyekum et al. [96] introduced an interpretable model using clinical and US features to predict ablation efficacy, while Liang et al. [97,98] demonstrated the use of deep convolutional neural networks (EfficientNet-based) trained on ultrasound images to predict nodules with suboptimal response (VRR < 50%) to microwave ablation, achieving high predictive accuracy (AUC = 0.85). These AI-driven approaches may offer non-invasive, image-based decision support systems that enhance patient stratification and allow more personalized treatment planning. While further prospective validation is needed, current evidence supports the integration of ultrasound-based risk factors and AI-assisted models into routine pre-ablation assessment to optimize safety and efficacy. Table 1 summarizes clinical outcomes and follow-up strategies in thyroid nodules ablation techniques.

## 8. Cost-Effectiveness of Thermal Ablation Techniques for Thyroid Nodules

Thermal ablation techniques radiofrequency ablation (RFA), microwave ablation (MWA), and laser ablation (LA) have emerged as cost-effective alternatives to surgical intervention for the management of thyroid nodules. A U.S.-based microsimulation model demonstrated that RFA is a dominant strategy compared to lobectomy, offering lower overall costs (~$16,563 vs. ~$19,262) and slightly improved quality-adjusted life years (QALYs), provided the procedural cost remains below $12,330 at a willingness-to-pay threshold of $50,000 per QALY [82]. Similarly, Miller et al. reported that RFA could reduce variable direct costs by up to 19%, identifying a break-even probe cost of approximately $2100 [83]. In a multicenter Chinese study, although RFA was associated with better short-term quality of life than open thyroidectomy, it was not deemed cost-effective due to higher procedural costs; however, the authors projected that a 30% reduction in equipment costs would improve its cost-effectiveness under local economic thresholds [84]. MWA has also demonstrated favorable economic outcomes, primarily by reducing hospital stay duration, eliminating the need for general anesthesia, and decreasing postoperative complications. Liu et al. highlighted that these factors contribute to significant cost savings without compromising clinical efficacy or patient satisfaction [85,86,87]. In a Chinese secondary hospital, MWA for benign thyroid nodules costs RMB 14,000 (≈US$2100), while for malignant nodules the cost ranges from RMB 20,000 to 30,000 (≈US$3000–4600) due to more extensive procedural requirements [90]. In that same setting, the disposable electrode used in MWA (or RFA) costs ~US$1600, accounting for over 60% of the total cost [91]. In Europe, cost components include the device itself (microwave generator + antenna system) and disposables. The association guidelines report that the capital cost of an MWA system is about €20,000–25,000, and the cost for a single disposable antenna (applicator) is roughly €1000–1250 [14]. LA has similarly shown substantial cost advantages, driven by reductions in hospitalization time, avoidance of general anesthesia, and low complication rates [81,88]. Pacella et al. emphasized LA’s high efficacy and safety profile, further enhancing its cost-effectiveness and patient acceptability [77]. Additionally, the outpatient nature of LA contributes to decreased indirect costs, such as reduced work absenteeism and faster return to normal activities [89]. In Europe, the cost of a disposable laser fiber kit typically ranges from €300 to €500 per procedure, while the capital cost of the diode laser generator is approximately €30,000 [14]. A recent Italian cost-analysis estimated the cost of using two laser fibers at €330 each, contributing to a total procedure cost of around €1560, which includes all materials, professional fees, and indirect costs [92]. Although initial equipment costs for all three modalities remain a consideration, increased procedural volumes and continued technological advancements are expected to further enhance their economic sustainability. Overall, current evidence supports thermal ablation techniques as clinically effective and economically viable alternatives to surgery in the treatment of thyroid nodules.

## 9. Conclusions

Radiofrequency ablation (RFA), microwave ablation (MWA), and laser ablation (LA) are effective, minimally invasive treatments for symptomatic benign thyroid nodules. These techniques reliably reduce nodule volume, alleviate compressive and cosmetic concerns, and improve quality of life. Each modality offers distinct technical advantages and maintains a favorable safety profile when performed under expert ultrasound guidance. Compared to surgery, thermal ablation offers key benefits, including outpatient treatment, avoidance of general anesthesia, and thyroid function preservation. Ongoing research is essential to refine patient selection, standardize protocols, and evaluate long-term outcomes. Thermal ablation is increasingly recognized as a central component in the conservative management of benign thyroid disease.

## Figures and Tables

**Table 1:** Comparison of clinical outcomes and follow-up strategies in thyroid nodule ablation techniques.

	RFA (Radiofrequency Ablation)	MWA (Microwave Ablation)	LA (Laser Ablation)
**Volume Reduction**	64.5% at 6 months [18] 76.9% at 1 year [22] 92.2% at 3 years [34] Mean: 72% [69]	45–65% at 12 months (general range) [47] Up to 90–94% [95]	68% at 1 year [18] 59% at 10 years [56] 47.8% at 3 years [71]
**Symptom Improvement**	High rates (>80%) of pressure and cosmetic relief [49] 84% [60]	Significant symptom relief reported [49] Similar to RFA and surgery (low-quality evidence) [60]	89% [49] 73% [60]
**Cosmetic Improvement**	72% [39] 81% [61]	Comparable to RFA (no major difference) [61]	71.3% [61]
**Regrowth Rate**	0–34% at 12 months [34] 24.1% regrowth at ~39 months [79]	Regrowth possible; repeat ablation may be needed [79]	1.7% at 4 years [79] 4.7% at 7 years [85]
**Influence of Nodule Type**	Cystic/spongiform nodules respond better [14] Solid nodules: slower, less reduction [18]	Mixed/predominantly cystic nodules reduce more due to fluid aspiration [18]	Not specified [18]
**Complication Rate**	Minor & major: 0.9% [69] Minor: 33% [71]	Generally low, but can include mild thyroid function changes (low TSH, high fT4) [71]	Minor only [71] Energy dose linked to complications [77]
**Thyroid Function** **Impact**	Generally preserved [63] Some normalization of TSH if VRR ≥80% [75]	Mild and temporary hormonal fluctuations reported [75]	Minimal impact; calcitonin monitored pre- and post-LA [75]
**Follow-Up Protocol**	US + Doppler at 1, 3, 6, 12 months [79] Symptoms & labs (TSH, FT4, antibodies) [83]	Same as RFA [83] Also monitor thyroid function if dysfunction symptoms occur [86]	US + Doppler at 3, 6, 12 months [83] Calcitonin checked pre-LA and 1 year post-LA [86]

## Data Availability

The authors confirm that the data supporting this study’s results are available within the present review.
